# Theoretical and experimental investigations of the CMOS compatible Pirani gauges with a temperature compensation model

**DOI:** 10.1038/s41378-024-00832-z

**Published:** 2025-01-23

**Authors:** Shizhen Xu, Gai Yang, Junfu Chen, Rui Jiao, Ruoqin Wang, Hongyu Yu, Huikai Xie, Xiaoyi Wang

**Affiliations:** 1 The School of Integrated Circuits and Electronics, Beijing Institute of Technology, 100081 Beijing, China; 2 The Mechanical and Aerospace Engineering Department, Hong Kong University of Science and Technology, Hong Kong, SAR, China; 3 BIT Chongqing Institute of Microelectronics and Microsystems, Chongqing, China

**Keywords:** Engineering, Electrical and electronic engineering

## Abstract

In this article, a CMOS-compatible Pirani vacuum gauge was proposed featuring enhanced sensitivity, lower detection limit, and high-temperature stability, achieved through the implementation of a surface micromachining method coupled with a temperature compensation strategy. To improve performance, a T-type device with a 1 µm gap was fabricated resulting in an average sensitivity of 1.10 V/lgPa, which was 2.89 times larger than that (0.38 V/lgPa) of a L-type device with a 100 µm gap. Additionally, FEA simulations were conducted, analyzing the influence of heater temperature on sensitivity and the attenuation of sensitivity across varying ambient temperatures. A semi-empirical theoretical mode was derived for performance prediction, demonstrating strong alignment with experimental results, underscoring its effectiveness in compensating for sensitivity attenuation. Building on the foundation, the device’s performance under different ambient temperatures was characterized and effectively compensated in two distinct operational modes: constant temperature mode and constant temperature difference mode (the whole range temperature compensation error can be controlled within 2.5%). Finally, the short-time stability (variation level is approximately 1 mV), noise floor (Vrms=384 μV) and detection limit (0.07 Pa @1 Hz) of the device were characterized, confirming its suitability for practical implementation.

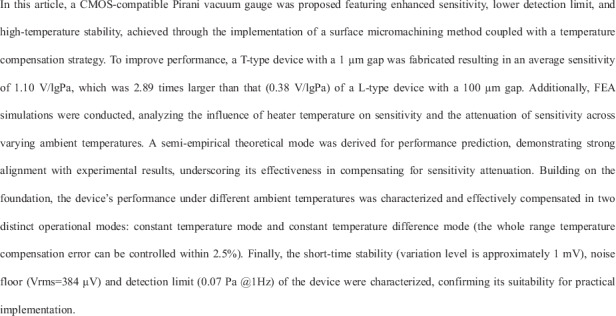

## Introduction

Vacuum detection plays an important role in the semiconductor manufacturing, medical, and automotive electronics industries. As the demand for lower power consumption, higher accuracy, and reduced costs increases, detection devices also require miniaturization. During this process, Pirani vacuum gauges have been widely studied in the field of MEMS due to their simple structure and compatibility with silicon-based semiconductor technology^1–7^. These gauges are typically used in conjunction with readout circuits to measure data. The CMOS compatibility in MEMS technology allows for the integration of Pirani vacuum gauges and readout circuits on the same silicon wafer.

Two important issues need to be addressed for Pirani vacuum gauges. One is improving sensitivity and output range, and the other is enabling the device to operate stably in various environments, such as different temperatures and gas conditions. There have been many studies on these two issues so far. Many studies improve performance by changing thermosensitive materials, such as polysilicon, vanadium oxide, titanium, platinum and nickel, which are commonly used heating resistors^8–13^. Guo et al.^14^ used vanadium oxide (VOx) film as a thermistor and utilized its high temperature coefficient of resistance (TCR) to improve output performance. The device has a dynamic range of 10^–1^ to 10^4 ^Pa and a sensitivity of 1.23 V/lgPa. For harsh environments, some special materials are also used. Mo et al.^15^ used Poly SiC material in the temperature range of 25 °C to 650 °C, and the pressure gauge has a repeatable response to pressure changes, with a Q/Pa of 1.23 at 650 °C. Zhang et al.^16^ proposed a Pirani device using nickel as the sensing material, which is vertically packaged with three silicon wafers through bonding. This structure can show good sensing performance under the pressure of 0.1-10 kPa. Furthermore, many researchers have established mathematical models for the impact of device parameters on output performance, and conducted experimental verification. Constant voltage (CV), constant current (CC), and constant temperature (CT) are three common power supply methods for heaters^17–19^. Santagata et al.^20^ derived the effects of output voltage, sensitivity, dynamic range, and device parameters of a Pirani bridge in CC mode, providing valuable insights for designers to balance performance, area, and cost. In the work of Xu et al.^21^, an empirical equation for the signal output in CT mode was proposed, which showed a good fit with experimental data. Song et al.^22^ extended this model and further applied it to the commonly used Wheatstone bridge-based readout circuit. Chen et al.^23^ prepared two types of Pirani devices with different gaps and sizes, using Ti as the sensing material. They combined the two measuring ranges by connecting them in series, and completed the power supply test through a constant bias voltage Wheatstone bridge circuit.

However, many devices’ fabrication processes are incompatible with CMOS, preventing further miniaturization and integration with on-chip circuits. Additionally, the reported work has seldom provided a specific analysis of the influence of ambient temperature on output performance or a strategy for temperature compensation.

In this paper, we present a CMOS-compatible technology to fabricate two miniature Pirani pressure gauges, featuring gap dimensions of 1 μm (T-type) and 100 μm (L-type), respectively. The T-type device was fabricated using surface sacrificial layer techniques, while the L-type device utilized deep silicon etching. We also derived a semi-empirical theoretical model to account for the influence of ambient temperature, enabling both comparative analysis and predictive modeling against experimental data. Furthermore, temperature compensation was performed using the enhanced model. The results indicate that the T-type device exhibits superior performance in terms of sensitivity and dynamic range, with the theoretical model demonstrating a strong correlation with experimental data under varying ambient temperature conditions. Additionally, the temperature compensation at room temperature proved to be effective. Finally, the short-time stability, noise floor, and detection limit of the device were characterized.

## Results and discussion

### Working principle

**Fig. 1:**
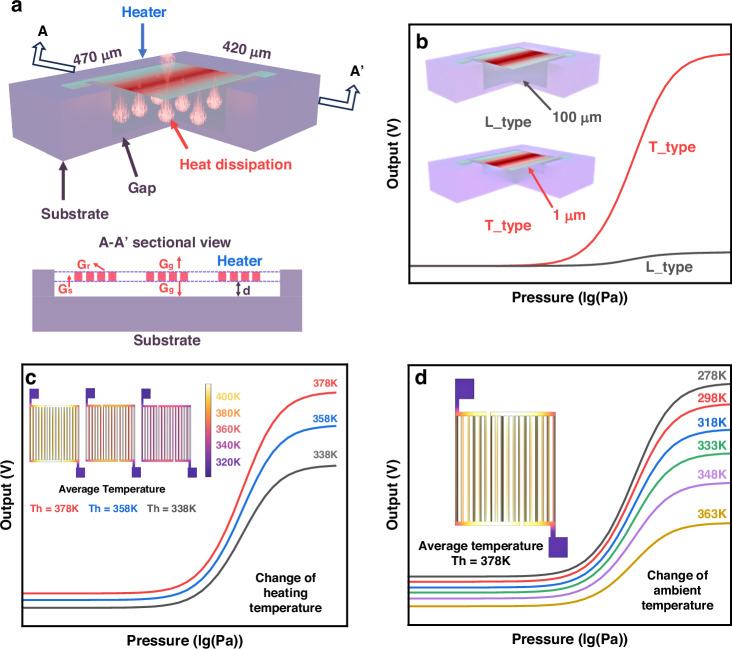
Schematic and basic simulation of Pirani vacuum gauge. **a** Schematic diagram of a micro Pirani vacuum gauge. **b** Simulation results of T-type and L-type devices. **c** Comparison of output performance at different operating temperatures. **d** Comparison of output performance under different ambient temperatures

In this paper, we designed CMOS-compatible micro Pirani vacuum gauges with two distinct gap distances, fabricated using sacrificial layer techniques and deep silicon etching, as illustrated in Fig. 1a. Pirani gauges generate electrical output by converting variations in vacuum levels into changes in thermistor resistance, which result from alterations in heat dissipation. The energy conservation equation for a micro Pirani vacuum gauge at steady state can be expressed as:

1 $${P}_{h}=({G}_{s}+{G}_{g}+{G}_{r})\varDelta T$$

2 $$\varDelta T={T}_{h}-{T}_{a}$$

where, P_h_ represents the heating power of the resistor, and *G*_*s*_, *G*_*g*_, and *G*_*r*_ are the solid thermal conductivity, gas thermal conductivity, and radiation thermal conductivity, respectively. *ΔT* is the temperature difference between the heating resistor *T*_*h*_ and the ambient temperature *T*_*a*_. These parameters can be specifically represented by the following equations:

3 $${P}_{h}=\,{U}_{h}^{2}/{R}_{h}$$

4 $${G}_{s}=\mathop{\sum }\limits_{i=1}^{n}\left({\lambda }_{i}\frac{{W}_{i}{H}_{i}}{{L}_{i}}\right)$$

5 $${G}_{g}=-\frac{{\lambda }_{g}\left(p,{T}_{a}\right)S}{d}$$

6 $${G}_{{rad}}=\frac{2\varepsilon {\sigma }_{b}{S}_{r}\left({T}_{h}^{4}-{T}_{a}^{4}\right)}{\varDelta T}$$

In these equations, *U*_*h*_ is the voltage across the heating resistor, *R*_*h*_ is the resistance of the heating resistor; *λ*_*i*_, *W*_*i*_, *H*_*i*_, and *L*_*i*_ are the thermal conductivity, width, height, and length of the solid material, respectively; ${\lambda }_{g}\left(p,{T}_{a}\right)$ is the thermal conductivity of the gas at pressure *p*, *S* is the sensing area, *d* is the gap distance; *ε* is the emissivity, *σ*_*b*_ is the Stefan-Boltzmann constant, *S*_*r*_ is the radiating surface area. In many studies, it has been mentioned that the radiative thermal conductivity is negligible in the operation mode of the micro Pirani vacuum gauge^17,21,22^, while the gas thermal conductivity *G*_*g*_ can be calculated as follows

7 $${\lambda }_{g}\left(p,{T}_{a}\right)={\lambda }_{0}\left. ({T}_{a}\right)/\left(1+2\left(\frac{2-a}{a}\right)\frac{l\left(p\right)}{d}\frac{9.5}{6}\right)$$

8 $${\lambda }_{0}\left({T}_{a}\right)=\,\mathop{\sum }\limits_{i=0}^{5}{\lambda }_{i}\,\cdot \,{T}_{a}^{i}$$

9 $$l\left(p\right)=\frac{k{T}_{a}}{\sqrt{2}\pi {\sigma }^{2}p}$$

where, *k* is Boltzmann’s constant, ${\lambda }_{0}\left. ({T}_{a}\right)$ is the thermal conductivity of air at atmospheric pressure at an ambient temperature of *T*_*a*_, a represents the energy accommodation coefficient for the molecules, *σ* represents the radius of an air molecule. It is worth noting that in many previous studies, the effect of ambient temperature on thermal conductivity was not calculated. Here, we have supplemented this by referring to the fitting formula for the thermal conductivity of air from Tsilingiris’s work^24^. Table 1 delineates the discrete numerical values attributed to each parameter.

**Table 1:** Parameter value^24^

Parameter	Quantity
*λ*_0_	–2.276501 × 10^–3^
*λ*_1_	1.2598485 × 10^–4^
*λ*_2_	–1.4815235 × 10^–7^
*λ*_3_	–1.73550646 × 10^–10^
*λ*_4_	–1.066657 × 10^–13^
*λ*_5_	2.47663035 × 10^–17^
*a*	0.77
*σ*	4 × 10^–10^

At the outset of our research, we conducted a preliminary analysis of the device’s performance through finite element simulations. A model was established and imported into Finite Element Analysis (FEA) simulation software, where physical field conditions were set to maintain the heating resistor at a constant temperature, allowing for the calculation of the corresponding voltage. The simulation results are shown in Fig. 1b–d.

As indicated by Eq. (5), a smaller gap leads to increased air thermal conductivity, thereby amplifying changes in heat dissipation with varying air pressure. To investigate this, we developed two structural configurations: T-type and L-type. Figure 1b illustrates the structures of these two devices utilized in the simulation. Finite element simulations reveal that the T-type device exhibits a broader output range and dynamic range under identical conditions. The relationship between the operating temperature of the device and the output voltage, as described by Eqs. (1)–(3), is also confirmed through simulation.

Additionally, to thoroughly analyze the effect of heater temperature on device performance, we conducted finite element simulations with varying operating temperatures for the constant temperature (CT) mode, as depicted in Fig. 1c. The heater section was set to average temperatures of 378 K, 358 K, and 338 K. The results indicate that the output voltage increases with the operating temperature, reflecting an increase in the fixed resistance of the CT mode. This, in turn, raises the working temperature of the heating element, thereby enhancing both sensitivity and output performance.

Furthermore, Eq. (1) illustrates that temperature difference has a substantial impact on the output voltage, a finding that is validated by simulations conducted at different ambient temperatures. To fully understand the effect of ambient temperature on device performance, we simulated various ambient temperatures ranging from 278 K to 363 K, with the working temperature fixed at 378 K, as shown in Fig. 1d. A notable observation is that the output voltage decreases significantly as the ambient temperature approaches the operating temperature. This sensitivity attenuation with increasing ambient temperature is attributed to the reduction in the overheat temperature (ΔT, the difference between the heater temperature and the ambient temperature) and the perturbations in gas properties^25^.

### Device fabrication

**Fig. 2:**
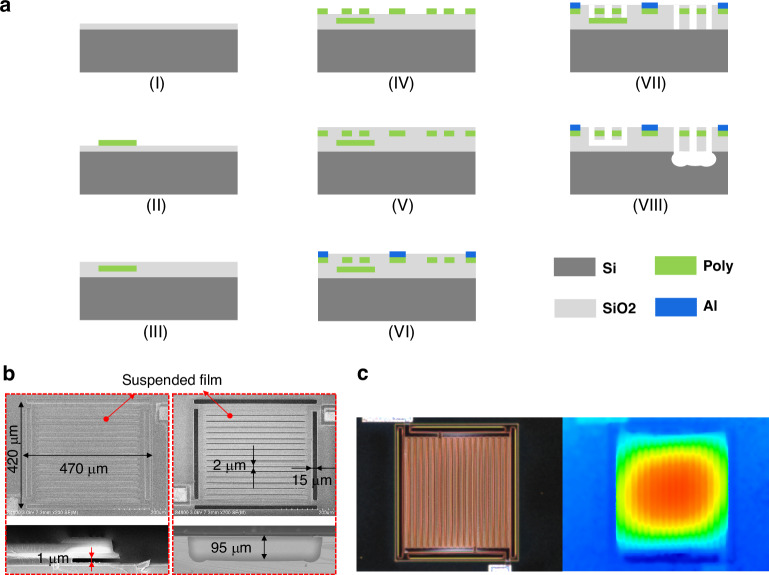
Process flow and morphology. **a** CMOS compatible fabrication process of the micro Pirani gauges. **b** SEM images of the fabricated Pirani gauge with tiny gap structure and large gap structure using the CMOS-compatible fabrication process. **c** Optical images and surface contour of T-type gauge

For device fabrication, we employed a CMOS-compatible process that can be integrated into a CMOS-MEMS design with on-chip circuits. Polysilicon was utilized as the thermistor material, and two distinct device structures were fabricated using sacrificial layer technology and deep silicon etching, as illustrated in Fig. 2.

Firstly, a 0.5 μm layer of silicon oxide was deposited on the surface of the wafer (Fig. 2a (I)). This was followed by the deposition of a 0.8 μm polysilicon sacrificial layer (Fig. 2a (II)). Next, a 1.5 μm silicon oxide layer was deposited as a support layer (Fig. 2a (III)), followed by the deposition of a 0.8 μm polysilicon thermistor part (Fig. 2a (IV)). A 1 μm silicon oxide layer was then deposited on top of the polysilicon structure as a protective layer (Fig. 2a (V)). Subsequently, a 2 μm aluminum layer was deposited to form the leads and pads (Fig. 2a (VI)). The silicon oxide was then selectively removed by reactive ion etching (RIE) to expose the silicon layer that needed to be etched away (Fig. 2a (VII)). Finally, the sacrificial layer and parts of the silicon substrate were etched using directional reactive ion etching (DRIE) and XeF2 methods to release the Pirani structure (Fig. 2a (VIII)).

In Fig. 2b, we present the SEM images of T-type and L-type devices, the top view SEM image shows the basic structure and dimensional details of the Pirani vacuum gauge, while the side interface image provides information about the gap. The image shows that the distance between the gaps basically conforms to our design (1 μm and 100 μm) during the fabrication process. Figure 2c demonstrates the optical micrograph of the Pirani vacuum gauge and the surface morphology of the device film after release. Due to the presence of stress, there are some bump deformations on the surface of the devices, which will directly affect the device’s spacing, the d in formulas 5 and 7, thus reducing the device’s output.

### Performance characterization

**Fig. 3:**
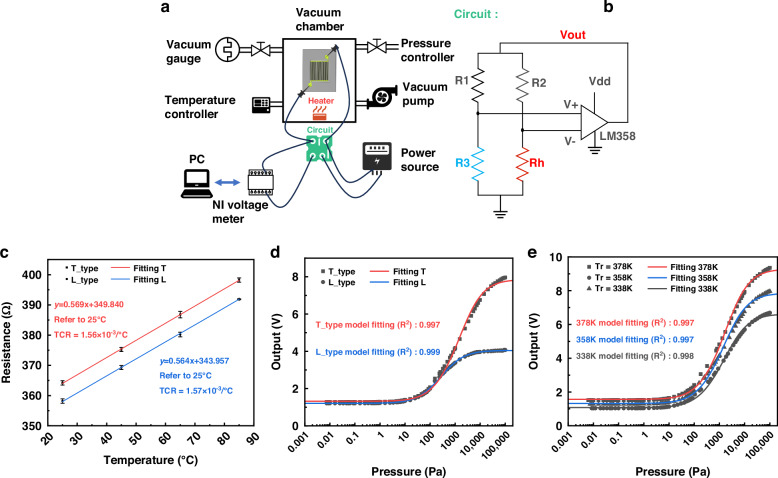
Test system and basic testing. **a** Schematic diagram of the test system. **b** Schematic diagram of the test circuits. **c** TCR characterization of the sensing material. **d** Test results and curve fitting of T-type and L-type devices. **e** Test results and curve fitting at different operating temperatures

The Pirani testing system (Fig. 3a) is designed to evaluate Pirani devices within a sealed chamber using a probing method. This system comprises a vacuum pump and pressure control system, as well as a heater and temperature control system, enabling simultaneous regulation of both pressure and ambient temperature. A constant voltage source supplies 15 V to the operational amplifier LM358. Pressure calibration is achieved using two commercial vacuum gauges: the Pfeiffer-Vacuum PKR251 and the Nanjing Hangjia Electronic Technology HPM18V. Control and measurement are conducted through a constant temperature circuit, with data acquisition and processing performed on a computer using an NI voltage acquisition card NI 9202.The circuit utilizes a bridge configuration and negative feedback to achieve constant temperature control (Fig. 3b). We set the ratio of R1 to R2 to 1:1. Due to the negative feedback, the resistance of the thermistor is maintained at R3, ensuring a constant operating temperature. In constant temperature (CT) mode, R3 is the fixed resistance. In constant temperature difference (CTD) mode, R3 is the series combination of the thermistor and the fixed resistance.

Prior to conducting the vacuum test, we initiated the process by examining the Temperature Coefficient of Resistance (TCR) for the two sensors. This was achieved by connecting the Pirani gauge leads to a Printed Circuit Board (PCB) and placing the sensors inside a constant temperature and humidity chamber (Espec SH222) to regulate temperature variations. Resistance data were recorded using a multimeter, and the procedure was repeated three times for each device. The data were then fitted, as shown in Fig. 3c. At a temperature of 25 °C, the TCR values for the two sensor structures were determined to be 1.56 × 10^–3^ per °C and 1.57 × 10^–3^ per °C, respectively.

First, test probes were conducted on sensors featuring distinct structures, with the ambient temperature maintained consistently while the operating temperature was set to 358 K. As clearly illustrated in Fig. 3d, the T-type sensor exhibited a dynamic measurement range from 0.16 Pa to 100 kPa, which is broader than the L-type’s range of 0.25 Pa to 70 kPa, and also showed a sensitivity that is more than double that of the other type. This specific content can be found in Figure S1a–c of the supplement material. To accurately represent the experimental data from both curves, we employed a semi-empirical formula:

10 $${U}_{{out}}=\sqrt{\left(a+\frac{b\,\cdot \,p}{p+c}\right)\left(\Delta T\right){\left({R}_{h}+{R}_{2}\right)}^{2}/{R}_{h}}$$

which was derived from theoretical considerations. The experimental results previously mentioned align with the simulation predictions, thereby confirming the superior output performance of T-type devices. Consequently, our subsequent research endeavors will concentrate primarily on T-type devices.

Then, the output performance of both was evaluated across various operating temperatures, and curve fitting was performed, as depicted in Fig. 3e. The results indicate that higher operating temperatures lead to an increase in sensitivity. Therefore, within an acceptable temperature range, we have chosen a higher temperature as the working condition.

**Fig. 4:**
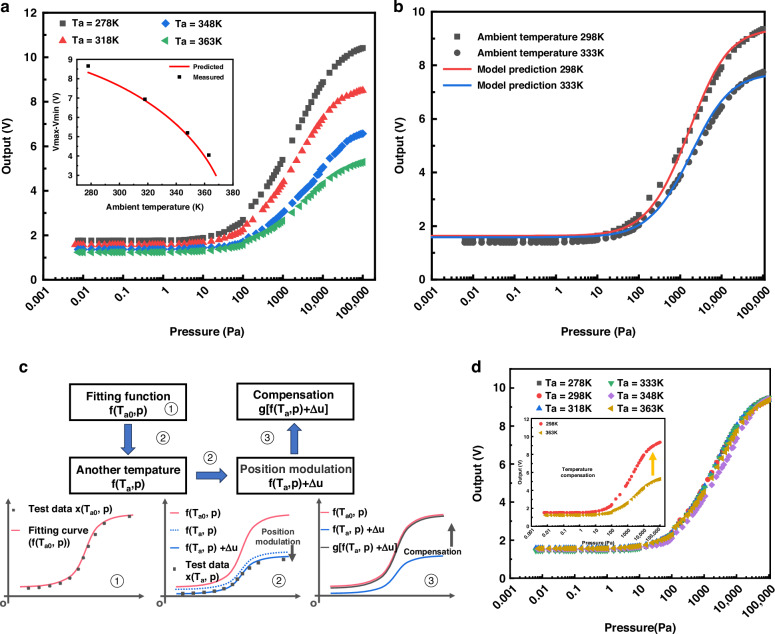
Temperature attenuation and temperature compensation. **a** Test results and theoretical model predictions of output range at different ambient temperatures. **b** Test results and theoretical model predictions at 298 K and 333 K. **c** Schematic diagram of temperature compensation process. **d** Temperature compensation based on an ambient temperature of 298 K

The working temperature was fixed at 378 K, and changes in ambient temperature were recorded, as depicted in Fig. 4a. As the ambient temperature nears the working temperature, the device’s sensitivity notably diminishes due to reduced heat dissipation, the variation range of the output signal at 363 K decreased by 53.3% compared to 278 K. The illustration highlights the impact of varying environmental temperatures on the output signal’s variation range, with black dots representing data points from four test curves and red curves showing predicted values. Using Eq. (10), we fitted the ambient temperature curve to room temperature conditions. We decomposed the temperature-dependent parameters *b* and *c* into their respective components:

11 $${b=\lambda }_{0}\left({T}_{a}\right)\,\cdot \,b^{\prime}$$

12 $$c{\,=T}_{a}\,\cdot \,c^{\prime}$$

where, ${\lambda }_{0}\left({T}_{a}\right)$ and *T*_*a*_ are functions of the ambient temperature *T*_*a*_. By substituting the value of ${\lambda }_{0}\left({T}_{a}\right)$ obtained from (8) into these expressions, we derived the values of *b*’ and *c*’. We input the experimental data into the semi-empirical fitting function to obtain the corresponding parameter values a, *b*’, *c*’. By inputting different *T*_*a*_ values, we predicted the effect of environmental temperature changes on output performance, which is visualized as the red line in the illustration. Calculating the degree of fitting yielded a fitting coefficient of 0.97, indicating a strong agreement between our theoretical model, especially noteworthy is its consideration of the impact of *T*_*a*_ on *λ*, along with the supporting experimental data.

We found that the predicted function is relatively accurate in predicting the relative waveform of the output signal, but there is still a deviation at the initial value, when the pressure is near zero. Knowing the output voltages at zero pressure and at atmospheric pressure, we use these as benchmarks to correct the predicted function. Specifically, we subtract the function value at zero pressure from the predicted function, and then add the actual test value at zero pressure to obtain a corrected function. Finally, we average these two corrected curves to obtain a refined prediction of the waveform at the given temperature, as shown in Fig. 4b.

13 $${U}_{{\!out}\left(298,p\right)}=\sqrt{\left({a}_{\left(298\right)}+\,\frac{{\lambda }_{0\left(298\right)}\,\cdot \,{b}_{(298)}^{{\prime} }\,\cdot \,p}{p+298\cdot {c}_{(298)}^{{\prime} }}\right)\left(378-298\right)\,\cdot \,\frac{{\left({R}_{h}+{R}_{2}\right)}^{2}}{{R}_{h}}}$$

14 $${U}_{{out}\left(298,p\right)}^{{\prime} }=\left({U}_{{out}\left({T}_{a},p\right)}+\Delta u\right)$$

15 $$\Delta u=\,{0.5}\left[{x}_{\left({T}_{a},{p}_{\min }\right)}-{U}_{{\!out}\left({T}_{a},{p}_{\min }\right)}+{x}_{\left({T}_{a},{p}_{\max }\right)}-{U}_{{out}\left({T}_{a},{p}_{\max }\right)}\right]$$

16 $${{U}_{{out}\left(298,p\right)}}=g\left[{U}_{{out}\left({T}_{a},p\right)}^{{\prime} }\right]$$

17 $$\left\{\begin{array}{c}g\left({U}_{{\!out}\left({T}_{a},p\right)}^{{\prime} }\right)=\,\sqrt{A+\frac{B\cdot C}{D+E}}\\ A=\,\frac{{\left({R}_{h}+{R}_{2}\right)}^{2}}{{R}_{h}}\cdot \left(378-298\right)\cdot {a}_{\left(298\right)}\\ B={\frac{A}{{a}_{\left(298\right)}}\cdot b}_{\left(298\right)}^{{\prime} }\cdot {\lambda }_{0\left(298\right)}\cdot {T}_{a}\\ {C}=\left({{U}^{\prime}_{\,out({T}_{a,p})}-\Delta{u}}\right)^{2}-{A}\frac{\left(378-{T}_{a}\right)}{(378-298)}\\ D=\frac{A\cdot \left(378-{T}_{a}\right)}{(378-298)}\cdot \left(\frac{{b}_{(298)}^{{\prime} }\cdot {\lambda }_{0\left({T}_{a}\right)}\cdot 298}{{a}_{(298)}}+\left(298-{T}_{a}\right)\right)\\ E=({T}_{a}-298)\cdot \left({U}_{{\!out}\left({T}_{a},p\right)}^{{\prime} }-\Delta u\right)^{2}\end{array}\right.$$

Subsequently, based on the previously described model, we performed temperature compensation using Fig. 4c. First, taking room temperature (298 K) as the reference, we performed fitting using the semi-empirical model shown in Eqs. (10)–(12), which assumes constant fitting parameters ***a***_**(298)**_, ***b*’**_**(298)**_, and ***c*’**_**(298)**_ (step ①). Next, we obtained the equations for different ambient temperatures by varying ${T}_{a}$. We observed a deviation between the current fitting data and the test data, so we adjusted the parameters using Eqs. (14)–(15), as shown in Fig. 4c (step②). Following this, we performed temperature compensation, aligning the equations for other ambient temperatures (${T}_{a}$) to the data at 298 K using Eqs. (16)–(17) (step ③). With the data transmitted to the backend processing module, in CT mode, we can also easily implement temperature compensation features. As shown in Fig. 4d, this compensation process aligns the waveforms, effectively illustrating our compensation method. The performance of our compensation is characterized by measuring the variation of the output voltage range (ΔV) with temperature, with the output range at room temperature (298 K, 7.738 V) serving as a reference. Over the tested temperature range (278-363 K), the output voltage ranged from a minimum of 7.616 V at 318 K to a maximum of 7.941 V at 333 K, representing relative changes of –1.58 to 2.63%.

**Fig. 5:**
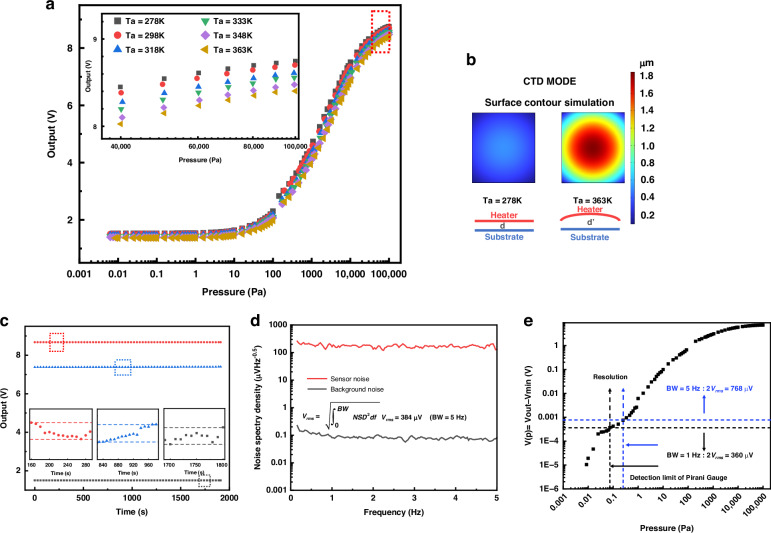
Performance characterization of CTD mode. **a** Test results at different ambient temperatures in the CTD operating mode. **b** Simulation of Surface Expansion in CTD mode. **c** Stability of the micro-Pirani vacuum gauge and test system. **d** NSD of the Pirani gauge and the background noise within a BW of 5 Hz. **e** The detection limit of the Pirani test system measured by *V*_*out*_*-V*_*min*_ and *V*_*rms*_

After thoroughly analyzing the impact of ambient temperature fluctuations, we also employed a constant temperature difference (CTD) circuit to compensate for these changes. Since both thermistors operate within the same ambient temperature, their actual operating temperature differential is maintained by a fixed resistor. The Pirani device was specifically set to operate at a constant temperature difference of 80 K above the ambient temperature. Under the same testing conditions, it was observed that the curves in Fig. 5a significantly mitigated the influence of ambient temperature variations. As shown in the illustration, there are slight differences in the output curve at different ambient temperatures, with the output signal variation range at 363 K decreasing by 2.5% compared to that at 278 K.

However, the results of the CTD method did not fully meet our initial expectations, as the output data exhibited lower values than anticipated when the ambient temperature increased. We speculate that this discrepancy could be due to a surface contour effect, as depicted in Fig. 2c, where thermal expansion of the film at higher temperatures leads to a slight increase in the gap, resulting in a decrease in the output voltage. As shown in Fig. 5b, we conducted a simplified simulation of surface expansion under the CTD mode. In this mode, the surface temperature of the film consistently remains 80 K higher than the ambient temperature. When the ambient temperature rises, a relatively pronounced expansion phenomenon occurs, which alters the gap distance and thus impacts the performance along with the predictive and compensatory effects of the formulas. The pre-existing deformation may intensify this effect. Referring to the method proposed by Garg et al., the actual gap value is calculated by bringing in deformation through averaging^18^. The calculation is carried out according to the following formula:

18 $${d}_{{eff}}=d+\frac{1}{S}\iint u\left(x,y\right)$$

where, u (x, y) is the contour deformation at point (x, y).

In addition, issues such as uniformity in the manufacturing process, the variation of solid heat conduction with pressure changes^26^, and the impact of environmental humidity on thermal conductivity^24,25^ during testing may all have a minor effect on our model. Taking these effects into consideration can make a certain degree of correction to our simulation and theoretical prediction in future studies.

As depicted in Fig. 5c, we conducted a 33-min stability test of the system under three distinct air pressure conditions. The illustration, which includes a partially magnified view, reveals that within a specific 2-min interval (inset figures of Fig. 5c) during the test, the system’s voltage varied by approximately 1 mV.

In Fig. 5d, the Noise Spectral Density (NSD) of both the system and the instrument itself was assessed utilizing a dynamic signal analyzer (Agilent 35670 A Dynamic Signal Analyzer). To characterize the noise issue, we chose two specific bandwidths: 1 Hz and 5 Hz. At these bandwidths, we calculated the Root Mean Square (RMS) noise, *V*_*rms*_, of the micro Pirani vacuum gauge, employing the integration formula indicated in the Fig. 5d. Subsequently, we defined a function ${V(p)=V}_{{out}}-{V}_{\min }$ and generated a graph with pressure plotted on the x-axis and $V(p)$ on the y-axis. To determine the detection range of the micro Pirani vacuum gauge, we used ${2V}_{{rms}}$ as the system’s resolution and drew a horizontal line at ${2V}_{{rms}}$ on the y-axis. The intersection point of this line with the test data curve indicates the lower limit of the gauge’s detection range. It can be concluded that the Pirani vacuum gauge is capable of achieving a detection limit of 0.07 Pa, as shown in Fig. 5e.

Table 2 presents a thorough analysis of the sensing capabilities of various Pirani sensors that have been developed. In comparison to these, our sensors, meticulously crafted using a CMOS-compatible MEMS process, display a notably advantageous detection limit. This attribute, along with their compatibility with CMOS technology, enables a smooth and cohesive monolithic integration with CMOS circuits. This integration capability is a significant advantage, making our sensors a strong candidate for future enhancements in sensor technology, particularly in terms of miniaturization and performance optimization

**Table 2:** Performances comparisons of the Pirani sensors

Ref.	Fabrication technologies	Temperature compensated	Pressure range (Pa)
Lee^8^	MEMS	No	6.65 ~ 10^5^
Guo^14^	MEMS	Yes (circuits)	0.1 ~ 10^4^
Zhang^16^	MEMS	No	0.1 ~ 10^4^
Chen^23^	MEMS	Yes (circuits)	0.066 ~ 1.12×10^5^
Zhang^6^	CMOS compatible	No	1.33 ~ 2.66×10^3^
Wang^7^	CMOS compatible	Yes	0.1 ~ 10^5^
Piotto^13^	CMOS compatible	No	0.3 ~ 10^5^
Garg^18^	CMOS MEMS	No	1 ~ 10^6^
Xu^21^	CMOS MEMS	No	0.067 ~ 10^5^
Song^22^	CMOS MEMS	No	0.8 ~ 1.4×10^5^
**This work**	**CMOS compatible**	**Yes(theory &circuits)**	**0.07** ~ **10**^**5**^

## Conclusion

In this article, we introduced a CMOS-compatible Pirani vacuum gauge with enhanced sensitivity, a lower detection limit, and high-temperature stability, achieved by implementing a surface micromachining method combined with a temperature compensation strategy. To improve performance, a T-type device with a 1 µm gap was fabricated, resulting in an average sensitivity of 1.10 V/lgPa, which is 2.89 times higher than that of an L-type device with a 100 µm gap. Additionally, to assess the impact of temperature on performance, Finite Element Analysis (FEA) simulations were conducted to analyze the influence of heater temperature on sensitivity and the attenuation of sensitivity across varying ambient temperatures. These simulations provided more accurate predictions and guided further experimental efforts.

A semi-empirical theoretical model was also developed for performance prediction, showing strong alignment with experimental results and achieving a fitting accuracy of 0.97. This underscores the model’s effectiveness in compensating for sensitivity attenuation. This model not only validates the design principles but also serves as a robust platform for future enhancements, enabling fine-tuning of device output to ensure consistent performance across varying conditions. Building on this foundation, the device’s performance under different ambient temperatures was characterized and effectively compensated using our proposed strategy in two distinct operational modes: constant temperature (CT) mode and constant temperature difference (CTD) mode (the temperature compensation error can be limited within 2.5%). This comprehensive analysis highlights the device’s potential for practical application, where maintaining accuracy across different thermal environments is crucial.

Finally, the device’s short-term stability, noise floor, and detection limit were thoroughly characterized to confirm its readiness for practical use. The short-time stability test revealed a voltage variation of 1 mV over a 2-min interval, demonstrating the device’s reliability in maintaining consistent output. Additionally, the noise floor was measured to be *V*_*rms*_ = 384 μV, and the detection limit was determined to be 0.07 Pa at 1 Hz. With its good performance and the ability to be integrated with CMOS characteristics, this device has a wider scope for future commercial applications.

## Materials and methods

### General characterization techniques

The instrumentation utilized in the Pirani testing system comprises a vacuum pump and pressure control system, a heater and temperature control system, a constant voltage source supplying 15 V to the LM358 operational amplifier, two commercial vacuum gauges (Pfeiffer-Vacuum PKR251 and Nanjing Hangjia Electronic Technology HPM18V) for pressure calibration, an NI voltage acquisition card NI 9202 for data acquisition and processing on a computer, a constant temperature circuit with a bridge configuration and negative feedback for temperature control, a Printed Circuit Board (PCB) for sensor connections, a constant temperature and humidity chamber (Espec SH222) for regulating temperature variations during TCR examination, and a multimeter for recording resistance data.

## Supplementary information

Theoretical and Experimental Investigations of the CMOS Compatible Pirani Gauges with a Temperature Compensation Model Supplemental Material
