# Supplementary material for: Theoretical and experimental investigations of the CMOS compatible Pirani gauges with a temperature compensation model
Source: Microsyst Nanoeng. 2025 Jan 23;11:21. doi: 10.1038/s41378-024-00832-z (PMC11754640; doi:10.1038/s41378-024-00832-z)
Supplement: Supplementary file 1 — Theoretical and Experimental Investigations of the CMOS Compatible Pirani Gauges with a Temperature Compensation Model Supplemental Material [file 41378_2024_832_MOESM1_ESM.docx]

**Supporting Information**

Theoretical and Experimental Investigations of the CMOS Compatible Pirani Gauges with a Temperature Compensation Model

*Shizhen Xu^1^, Gai Yang^1^, Junfu Chen^1^, Rui Jiao^2^, Ruoqin Wang^2^, Hongyu Yu^2^, Huikai Xie^1,3^*, Xiaoyi Wang^1,3^**

1 the School of Integrated Circuits and Electronics, Beijing Institute of Technology, Beijing, 100081, China.

2 the Mechanical and Aerospace Engineering Department, Hong Kong University of Science and Technology, Hong Kong, SAR, China.

3 BIT Chongqing Institute of Microelectronics and Microsystems, Chongqing, China.

**Corresponding authors:**

Prof. Huikai Xie

Email: [hk.xie@bit.edu.cn](mailto:hk.xie@bit.edu.cn)

Telephone: +86- 010-68918442

Prof. Xiaoyi Wang

Email: [xiaoyiwang@bit.edu.cn](mailto:xiaoyiwang@bit.edu.cn)

Telephone: +86-15868802615


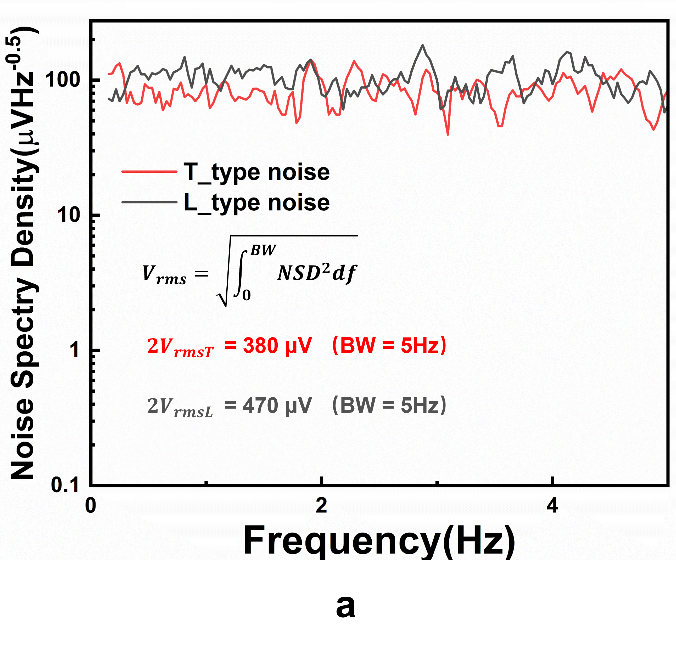


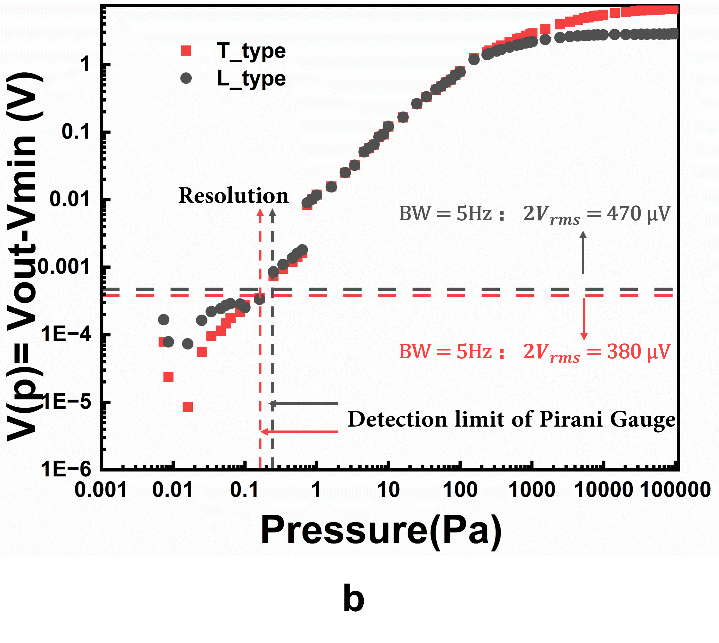


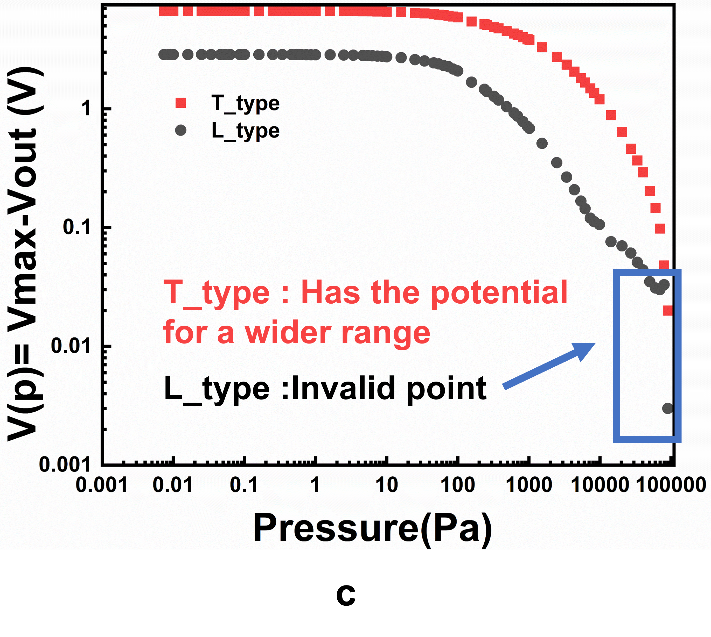


**Fig. S1 a** NSD of the Pirani gauges within a BW of 5Hz. **b** The detection limit of the Pirani test system measured by $V_{out}-V_{min}$ and $V_{rms}$. **c** The detection limit of the Pirani test system measured by $V_{max}-V_{out}$.
